# Analysis of selection signatures on the Z chromosome of bidirectional selection broiler lines for the assessment of abdominal fat content

**DOI:** 10.1186/s12863-021-00971-6

**Published:** 2021-05-31

**Authors:** Tao Wang, Meng Zhou, Jing Guo, Yuan-Yuan Guo, Kun Ding, Peng Wang, Zhi-Peng Wang

**Affiliations:** 1 Key Laboratory of Chicken Genetics and Breeding, Ministry of Agriculture and Rural Affairs, Harbin, China; 2 Key Laboratory of Animal Genetics, Breeding and Reproduction, Education Department of Heilongjiang Province, Harbin, China; 3 College of Animal Science and Technology, Northeast Agricultural University, Harbin, China; 4 Bioinformatics Center, Northeast Agricultural University, Harbin, China; 5 Key Laboratory of Healthy Mariculture for the East China Sea, Ministry of Agriculture and Rural Affairs, Jimei University, Xiamen, China; 6 College of Computer Science and Technology, Inner Mongolia Normal University, Huhehot, China; 7 HeiLongJiang provincial Husbandry Department, Harbin, China

**Keywords:** Z chromosome, SNP, Selection signature, Population genetics, Gene expression

## Abstract

**Background:**

The discovery of selection signatures has enabled the identification of genomics regions under selective pressure, enhancing knowledge of evolutionary genotype-phenotypes. Sex chromosomes play an important role in species formation and evolution. Therefore, the exploration of selection signatures on sex chromosomes has important biological significance.

**Results:**

In this study, we used the Cross Population Extend Haplotype Homozygosity Test (XPEHH), F-statistics (F_ST_) and EigenGWAS to assess selection signatures on the Z chromosome in 474 broiler chickens via Illumina chicken 60 K SNP chips. SNP genotype data were downloaded from publicly available resources. We identified 17 selection regions, amongst which 1, 11 and 12 were identified by XPEHH, F_ST_, and EigenGWAS, respectively. Each end of the Z chromosome appeared to undergo the highest levels of selection pressure. A total of 215 candidate genes were located in 17 selection regions, some of which mediated lipogenesis, fatty acid production, fat metabolism, and fat decomposition, including *FGF10, ELOVL7*, and *IL6ST*. Using abdominal adipose tissue expression data of the chickens, 187 candidate genes were expressed with 15 differentially expressed genes (DEGs) in fat vs. lean lines identified. Amongst the DEGs, *VCAN* was related to fat metabolism. GO pathway enrichment analysis and QTL annotations were performed to fully characterize the selection mechanism(s) of chicken abdominal fat content.

**Conclusions:**

We have found some selection regions and candidate genes involving in fat metabolism on the Z chromosome. These findings enhance our understanding of sex chromosome selection signatures.

**Supplementary Information:**

The online version contains supplementary material available at 10.1186/s12863-021-00971-6.

## Background

The domestication of chickens in Asia (*Gallus gallus*) occurred around 5400 BC with Darwin suggesting their evolution from red jungle fowl [1, 2]. Chickens hold value from an evolutionary perspective as they provide information that bridges knowledge between mammals and other vertebrates [3]. Domestic chickens have genetically adapted to unique habitats through strong genetic and phenotypic alterations. To date, an array of specialized commercial populations and inbred chicken lines have subsequently been developed.

Selection has many effects on the genome. Allele frequencies and polymorphism underlying selection are expected to change. With the availability of high-quality draft sequences of the chicken genome, high-density single nucleotide polymorphism (SNP) genotyping chips, and whole-genome re-sequencing technologies, the detection of selection signatures on the chicken genome have been reported. Rubin et al. [4] identified the *TSHR* gene (thyroid stimulating hormone receptor) as a prominent selection signature in all domestic chickens. Guo et al. [5] identified 413 candidate genes in Xishuangbanna fighting chickens that were related to aggressive behavior, including *BDNF, NTS* and *GNAO1*. Boschiero et al. [6] revealed more than 300 regions of selection with many important genes, including *AKAP6, IGFBP2* and *IGF1R*, associated with fat deposition and muscle development.

Sex chromosomes play an important role in species formation and evolution. Mcvicker et al. [7] analyzed the selective forces that shape hominid evolution and found that under natural selection, the selection pressure of sex chromosomes (12–40%) exceeded those of the autosome (19–26%). The selection pressure of autosomes and sex chromosomes is different, and when considering sex-specific dosage compensation, genes on the sex chromosomes are more directly and efficiently selected than those on autosomes [8, 9]. The size of the chicken Z chromosome is approximately 83 Mb, accounting for 7.9% of the chicken genome. The Z chromosome contains 1345 genes, and some genes, including *FGF10* (fibroblast growth factor 10), *ELOVL7* (ELOVL fatty acid elongase 7) and *ACO1* (aconitase 1, soluble), regulated fat deposition and development. Previous studies have focused on the selection signatures of chicken autosomes, but the selection signals of the chicken Z chromosome less well studied. Zhang et al. [10] only identified *PC1/PCSK1* gene, located on the Z chromosome, related to abdominal fat traits used selection signals and genome-wide association analysis based on NEAUHLF (Northeast Agricultural University broiler lines divergently selected for abdominal fat content) population. It is therefore necessary to identify as many selection signatures as possible on the Z chromosome in chickens.

In this study, we used the XPEHH, F_ST_ and EigenGWAS methods to identify the selection signatures associated with abdominal fat in the Z chromosomes of NEAUHLF populations. Through the integration of gene microarrays in the adipose tissue of NEAUHLF populations, we investigated the expression profiles of the candidate genes on selection regions at 7 weeks of age. Gene annotations and functional enrichment were implemented to elucidate the significance of the identified selection signatures to fat containing traits.

## Results

### Population structure

**Fig. 1:**
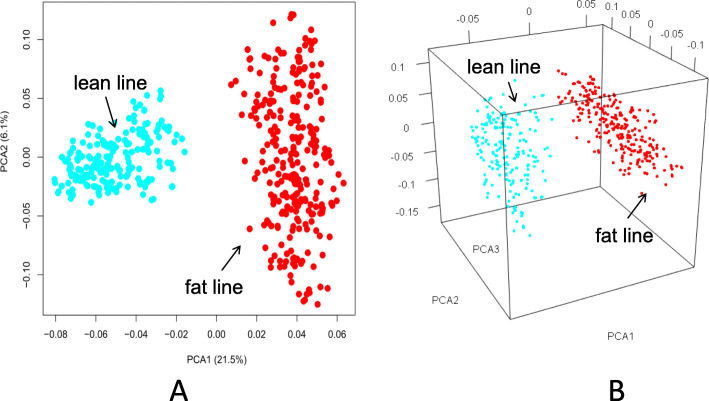
Population structure based on Z chromosome SNPs using principal component analysis. The subgraph (**a**) and (**b**) represent two-dimensional and three-dimensional PCA images

We performed principal component analysis (PCA) on 1937 SNPs on the Z chromosome to identify individual patterns. The first principal component (21.5% of the total variance) could separate the two lines (Fig. 1a). The second principal component (6.1% of the total variance) primarily revealed genetic differences in the fat lines, whilst the third principal component (5.3% of the total variance) primarily revealed differences in the lean lines (Fig. 1b).

### Selection signatures on the Z chromosome

**Fig. 2:**
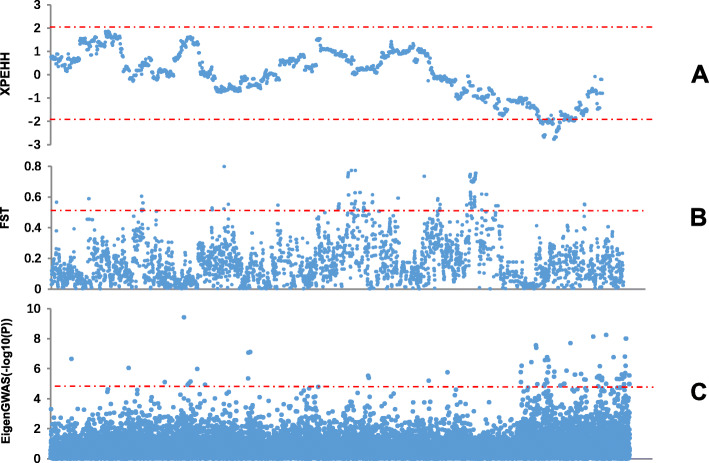
Selection signatures on the Z chromosome. The subgraphs (**a**), (**b**) and (**c**) are the selection signatures detected between the populations using the XPEHH, F_ST_ and eigenGWAS methods, respectively

Chicken Z chromosome selection signatures were identified between fat and lean line populations. Table 1 summarizes the selection signatures obtained using XPEHH, F_ST_ and EigenGWAS. For the fat-lean line pair, 1, 11 and 12 selection regions were identified using XPEHH, F_ST_ and EigenGWAS methods, respectively (Table 2 and Fig. 2). A total of 17 candidate regions were identified by merging these regions. The majority of the identified selection regions were present on both ends of the Z chromosome, accounting for about 53%. Amongst them, one candidate region (61.68–73.63 Mb) was identified by all methods, and five candidate regions were identified by F_ST_ and EigenGWAS. There were 5 and 6 candidate regions only identified by F_ST_ and EigenGWAS method, respectively.

**Table 1:** Selection signatures in the two chicken lines

Items	Fat - Lean		
	XPEHH	F_ST_	EigenGWAS
Number of significant SNPs	50	98	83
Number of regions	1	11	12
Average length (Mb)	2.24	0.63	1.71
Total length (Mb)	2.24	6.88	20.53

**Table 2:** Selection regions on the Z chromosome and candidate genes detected in the regions

Candidate Region (Mb)	F_ST_				EigenGWAS				XPEHH			
	Region (Mb)	Top snp	F_ST_	Major Genes	Region (Mb)	Top snp	*P*-value	Major Genes	Region (Mb)	Top snp	XP EHH	Major Genes
0.55–0.75	0.55–0.75	rs14688003	0.56	*WDR7*								
1.91–2.31					1.91–2.31	rs312273884	2.24E-07	*SLC14A2*				
4.53–4.66	4.53–4.66	rs16704177	0.59									
6.95–7.35					6.95–7.35	rs14689552	2.40E-05	*KIAA1328*				
9.25–9.65					9.25–9.65	rs14784526	9.10E-07	*ARHGEF39*				
11.23–11.57	11.23–11.57	rs16781096	0.57	*SLC1A3,*								
13.95–14.35					13.95–14.35	rs16103326	7.97E-06	*FGF10*				
16.75–19.95	19.81–19.95	rs14753903	0.52		16.75–17.42	rs16760225	7.23E-06	*IL6ST*				
					17.80–18.20	rs16099146	1.05E-06	*PLK2*				
					18.71–19.11	rs736458094	1.18E-05	*ELOVL7*				
21.22–23.50	21.22–21.84	rs14755170	0.66	*MAST4*	22.90–23.50	rs313728591	7.91E-08	*IQGAP2*				
28.08–28.19	28.08–28.19	rs16106682	0.55	*KDM4C*								
32.13–33.63					32.13–32.53	rs16108416	2.11E-05	*BNC2*				
					33.03–33.63	rs314666735	1.66E-05	*ADAMTSL1*				
36.03–40.49	36.03–36.25	rs16081040	0.54	*ANXA1*	39.07–39.87	rs16781643	3.03E-06	*FRMD3*				
	37.29–37.85	rs16768474	0.68	*VPS13A*								
	38.04–38.89	rs13817231	0.59	*TLE4*								
	39.22–39.45	rs14787078	0.54									
	40.33–40.49	rs14787751	0.57	*NTRK2*								
47.72–51.04	47.72–47.58	rs15597824	0.59	*FER*	48.33–51.04	rs314662658	6.33E-06	*EFNA5*				
	50.89–51.02	rs14766378	0.73	*FAM174A*								
52.48–53.77	52.50–52.89	rs16770232	0.53	*SLC49A3*	52.48–52.88	rs14768956	1.78E-06	*PCGF3*				
					53.37–53.77	rs14769714	2.52E-05	*ATP5I*				
55.98–60.28	55.98–57.39	rs14751311	0.66	*PCSK1*								
	58.12–58.85	rs16758604	0.60	*NR2F1*								
	59.90–60.28	rs14748688	0.54	*CETN3*								
61.68–73.63	71.43–71.58	rs14781326	0.55	*PRR16*	61.68–62.12	rs14774149	6.47E-07		65.73–67.97	rs14776247	−2.78	*DNAJC25*
					62.78–63.58	rs14773275	3.68E-07	*VCAN*				
					63.88–64.28	rs16118182	2.81E-08	*CKMT2*				
					64.82–66.08	rs14774834	1.72E-07	*SLC46A2*				
					66.24–66.67	rs13788226	2.66E-07	*DNAJC25*				
					66.84–67.88	rs16775262	1.59E-06	*ELAVL2*				
					68.59–68.99	rs16776912	2.02E-08	*MOB3B*				
					69.41–70.09	rs14780063	2.38E-05					
					70.35–71.36	rs16125209	2.28E-06	*ACO1*				
					72.16–73.63	rs14781762	7.41E-09	*KCNN2*				
78.84–82.42					78.84–79.27	rs15992604	5.72E-09	*CDKN2A*				
					79.67–80.45	rs14685714	1.75E-07	*ZNF608*				
					80.62–82.42	rs16683478	9.91E-09	*PAX5*

### Candidate gene annotations for functional analysis

According to the chicken gene annotation data (*Gallus gallus 6.0*) in the ENSEMBL database, we detected 215 candidate genes within 17 selection regions. Supplementary Table 1 summarizes the genes in each selection region on the Z chromosome. A number of genes were found to regulate lipogenesis, fatty acid production, fat metabolism, or fat decomposition, including *FGF10, ELOVL7* and *IL6ST*.

To reveal the biological functions of the genes within the identified regions, gene Ontology (GO) pathway enrichment analyses were performed using DAVID (v6.7) [11]. Significant GO functional terms (*P* < 0.05) are listed in Table 3, but these terms were not significant upon Benjamini correction.

**Table 3:** Functional enrichment analysis of the selected genes

Category	GO ID	Term	*P* value
Biological Progresses	GO:0043171	peptide catabolic process	0.0195
Cellular Components	GO:0005892	acetylcholine-gated channel complex	0.0231
	GO:0045211	postsynaptic membrane	0.0204
Molecular Function	GO:0070006	Metalloaminopeptidase activity	0.0154
	GO:0042166	acetylcholine binding	0.0230
	GO:0004889	acetylcholine-activated cation-selective channel activity	0.0230

A total of 1229 QTLs were found on the chicken Z chromosome in the QTLdb database (https://www.animalgenome.org/cgi-bin/QTLdb/GG/index). The selection signatures overlapped on 132 QTLs of health, physiology, exterior and production categories. Interestingly, 45 of the candidate genes that overlapped with the QTL region were related to abdominal fat weight, 22 were related to liver weight, 12 were related to food intake, 11 to residual food intake, and 18 to food conversion ratios.

### Expression profiles of the candidate genes in the selection regions

**Fig. 3:**
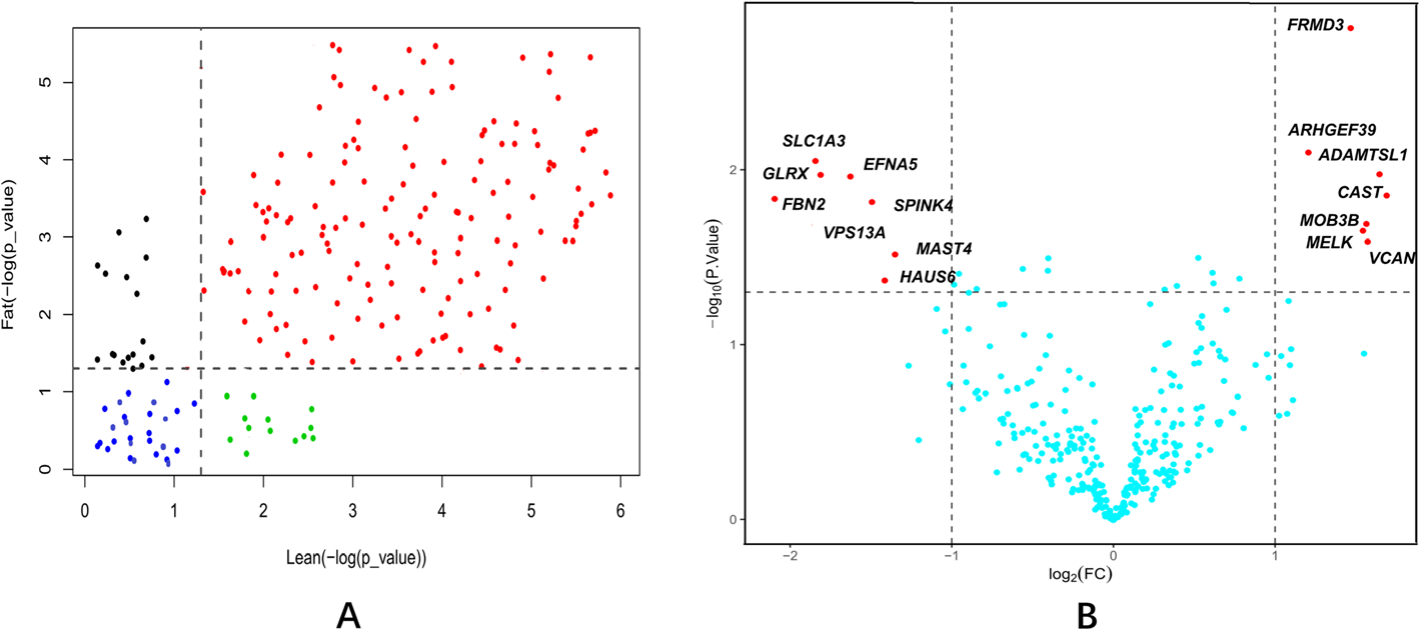
Candidate gene expression profiles in the adipose tissue of the NEAUHLF. The subgraph (**a**) is the gene expression profile of two chicken strains. The x-axis and y-axis is - log (*P*_value) of lean and fat line respectively, and the threshold value was *p* < 0.05. The red points indicate 155 genes that are expressed in the abdominal fat of both thin and fat lines, while the blue points indicate 28 genes that are not expressed in either line. The 13 green points indicate genes expressed in the lean line but not in the fat line and vice versa for the 19 black points.. The subgraph (**b**) shows the differential expression of genes in adipose tissues in fat and lean lines at the 7th week of age. The threshold is *P* < 0.05, fold change > 2. The red points indicate genes that are differentially expressed in the two chicken lines

We extracted probe sets of the Gene Chip Chicken Genome Array to represent all candidate genes located on the selection regions. A total of 427 probes representing 187 of the 215 candidate genes were identified. As shown in Fig. 3, 168 and 174 genes (*p* < 0.05) were expressed in lean and fat chicken lines at 7 weeks, respectively (Fig. 3a), whilst 15 DEGs (*P* < 0.05, fold change > 2) between fat and lean lines were identified (Fig. 3b). Seven genes showed significantly higher expression in the fat line (e.g. *ADAMTSL1, FRMD3, MOB3B, ARHGEF39, VCAN, CAST* and *MELK*) and eight genes in the lean line (e.g. *HAUS6, MAST4, VPS13A, SPINK4, SLC1A3, GLRX, FBN2* and *EFNA5*).

## Discussion

High-density SNPs chips permit the identification of genome-wide selection signatures using site frequent spectrums, population differentiation, and linkage disequilibrium, with known strengths and weaknesses. In this study, we used three complementary statistical approaches (F_ST_, XPEHH, EigenGWAS) to explore the selection signatures on the Z chromosome to minimize bias and false positives in the broiler chickens. The F_ST_ method is best suited for the detection in events occurring in the more distant past [12]. The F_ST_ method is a powerful tool to detect signatures based on group differentiation. The XPEHH test compares extended haplotype homozygosity between populations to detect selection signatures, which are segregated in populations and represent points of ongoing selection. XPEHH is therefore useful for the detection of entirely or approximately fixed loci [13]. The XPEHH test is an LD-based method, and LD is expected to extend over longer distances in regions under recent selection. So, selection regions detected by XPEHH were much wider [14]. Ma et al. [15] pointed out that the F_ST_ method may bring a higher false positive rate compared with XPEHH. The EigenGWAS algorithm combines the statistical framework of GWAS with eigenvector decomposition to identify selection signatures in the genomes of the underlying population. The EigenGWAS method uses multi-point information to identify core SNPs and grid windows, and can identify potential loci during selection, and a larger number of selection regions than F_ST_ and XPEHH [16]. Due to the similarities and differences principles between F_ST_, XPEHH and EigenGWAS, there are differently selection regions can be obtained using the different statistical approaches.

The sex (X) chromosome undergoes more drift than autosomes, as its effective population size (Ne) is three-quarters that of autosomes [17]. McVicker et al. [7] found that X chromosome has suffered higher selection pressure than autosomes. The NEAUHLF broiler population came from bi-directional divergently selected for abdominal fat content. Zhang et al. [10] found that four candidate regions of chromosome Z were identified as selection signature using long-range heterozygosity changes or allele frequency differences methods, and the 0.73 Mb *PC1/PCSK1* region of the Z chromosome was the most heavily selected region based on genome-wide using the NEAUHLF populations. The Z chromosome contains some genes involving fat metabolism, such as *FGF10*, *ELOVL7* and *ACO1.* However, Zhang et al. [10] did not identify selection signatures overlapped these genes. Selection signatures determined by multiple methods are deemed more credible [15, 18]. So, in this study, we used more methods to independently identify potential selection regions on the Z chromosome related on abdominal fat development to verify and supplement the previous findings. We detected three regions overlapped Zhang’s results using the F_ST_ method based on population differentiation (lean vs fat lines) or the EigenGWAS method in this study. Furthermore, we identified 14 other selection regions. These novel selection regions will provide specific gene targets for the control of chicken fatness traits or other traits genetically correlated with fatness. For example, we identified 61.68–73.63 Mb regions detected by three methods, and 69 genes that overlapped with the region, including *DNAJC25*, *GNG10* and *AKAP2*. Interestingly, *DNAJC25* is a member of DNAJ gene family identified by Liu et al. [19] as highly expressed in chicken liver tissue using transcriptome sequencing analysis. The *DNAJB6* gene, located on gga2, is a member of the DNAJ gene family and has a similar sequence to the *DNAJC25* gene. Jin et al. [20] previously found that the *DNAJB6* gene was expressed in the abdominal fat and liver tissues of the 14th generation NEAUHLF population, and was differentially expressed between the fat line and the lean line. Moreover, the expression level of *DNAJB6* in abdominal adipose tissue was significantly negatively correlated with abdomen fat weight and abdomen fat percentage [20].

In this study, there are 215 candidate genes overlapped 17 selection regions on chromosome Z. Amongst the candidate genes, *IL6ST, ELOVL7, CKMT2* and *FGF10* genes were also identified by Gholami et al. [21] in three commercial layer breeds and 14 non-commercial breeds. The *VCAN, ST8SIA4, FBN2, ERAP1* and *CAST* were also identified by Fu et al. [14], showing 10 regions of high confidence for selection on the Z chromosome, detected in male Cornish lines (a meat type breed), and female lines from White Rock (a dual-purpose breed). We also found that the majority of candidate genes expressed in the adipose tissue of G8 NEAUHLF fat and lean lines, and 15 genes (including *MAST4, SPINK4, VCAN, EFNA5, ARHGEF39,* etc) in the adipose tissue significantly differed between fat vs. lean birds using microarray gene expression data. Among them, *MAST4* encodes a microtubule-associated serine/threonine kinase; *SPINK4* is a serine peptidase inhibitor; and *ARHGEF39* encodes a rho guanine nucleotide exchange factor key to Rho mediated signal transduction.

According to known gene functions, some candidate genes were associated with the fat content of chickens, such as the *FGF10* gene. *FGF10* is a mesenchymal factor affecting epithelial cells. Matsubara et al. [22] reported that *FGF10* when secreted in chicken adipose tissue contributes to adipogenesis, and is down regulated during the early stages of chicken adipocyte differentiation. Konishi et al. [23] showed that *FGF10* stimulates proliferation in the white adipose tissue of mice. In addition, Yamasaki et al. [24] highlighted *FGF10* as an important intercellular signaling molecule during lipogenesis that is abundantly expressed in the adipose tissue of adult rats.

Due to high species conservation, the identified genes related to human or mice obesity traits may hold importance for adipose deposition in chickens, such as *ELOVL7, IL6ST, IQGAP2, PAX5* and *CKMT2* (Table 4). *ELOVL7* shows altered affinity for the elongation of precursor fatty acids and mediates the extension of saturated fatty acids of up to 24 carbon atoms [26]. *IL6ST* is an IL-6 transducer and a potent modulator of fat metabolism in humans, known to increase fat oxidation and fatty acid re-esterification [25]. *IQGAP2* deficiency influences hepatic free fatty acid uptake, fatty acid synthesis, and lipogenesis, suggesting its importance in obesity [27]. *PAX5* is a paired box 5 gene for which Melka et al. [29] performed GWAS in human adolescents from the French-Canadian founder population, revealing the association of its locus with total fat mass (TFM) and body mass index (BMI) in 6.4 and 3.7% of TFM and BMI heritability estimates, respectively. These results imply that *PAX5* plays a key role in obesity regulation. *CKMT2* (creatine kinase, mitochondrial 2) is a creatine kinase isoenzyme. Müller et al. [28] showed that *CKMT2* is an effective modulator of ATP synthase coupled respiration and is exclusively expressed in human brown adipose tissue. *CKMT2* also regulates energy metabolism.

**Table 4:** Candidate genes in the selection regions and their functions

Gene symbol	Location (Mb)	Full name	Function of association
*FGF10*	13.97–14.03	Fibroblast growth factor 10	Promotes fat formation [22]
*IL6ST*	17.02–17.06	Interleukin 6 signal transducer	Increases fat oxidation and fatty acid re-esterification [25].
*ELOVL7*	18.87–18.91	ELOVL fatty acid elongase 7	Extension of saturated fatty acids [26]
*IQGAP2*	23.46–23.58	IQ motif containing GTPase activating protein 2	Influences the liver uptake of free fatty acids, fatty acid synthesis and adipogenesis [27]
*CKMT2*	63.95–63.98	Creatine kinase, mitochondrial 2	Regulation of energy metabolism, expression in brown adipose tissue [28]
*PAX5*	82.01–82.12	Paired box 5	Regulates obesity [29]

## Conclusion

In this study, 17 selection regions were screened through the analysis of selection signatures in the chicken Z chromosome, including 215 candidate genes, some of which are involved in lipogenesis, fatty acid production, fat metabolism and fat decomposition, such as *FGF10, ELOVL7, IL6ST*. Moreover, in the candidate region, using abdominal fat expression data from chickens, 187 candidate genes were identified as expressed in the fat and lean lines, with 15 genes identified as differentially expressed. GO pathways enrichment and QTL annotations provided additional information on the selection mechanism(s) of chicken abdominal fat content. The culmination of these data enhances our understanding of sex chromosome selection signatures and their role in fat deposition in chickens.

## Methods

### Genotype data and population

SNP genotype data were downloaded from GEO Datasets on the NCBI website (GEO accession: GSE58551) [30]. Based on Illumina chicken 60 K SNP chips, 48,035 SNPs from 28 autosomes and Z chromosomes in 475 male broilers of the 11th generation (G11) (203 lean lines and 272 fat lines) were identified from NEAUHLF [10]. We mapped the SNP loci on the Z chromosome of all birds to the chicken reference genome (*Gallus gallus* 6.0), resulting in 1973 SNP loci. We applied QC measurements on the SNP loci on the Z chromosome of all birds using PLINK (v1.90) software: (1) SNP loci call rates of 0.95; (2) Sample call rates of 0.95; and (3) Minor allele frequencies (MAF) of 0.01 were discounted. In total, 1937 SNPs and 474 birds were investigated to detect selective sweeps in the chicken sex chromosomes. Specific details of broiler breeding strategy have been described by Zhang et al. [10].

### Principal component analysis

We performed PCA to distinguish population structures using EigenGWAS software [16] based on 1937 SNPs on the Z chromosome. The first ten eigenvalues and their corresponding eigenvectors were then calculated.

### Detection of selection signatures

Extended haplotype homozygosity (EHH) scores measure the probability that two randomly selected chromosomes carry a tested core haplotype that is homozygous at all SNPs [31]. XPEHH scores can detect selective sweeps in which a selected allele has achieved fixation in one population but remains polymorphic in another [32]. F_ST_ can identify genomic regions with strongly differing or differentially fixed variants in alleles frequency between different populations, which is the conventional measure of population genetic differentiation. F_ST_ is defined as follows:

$$ {F}_{ST}=\frac{MSP- MSG}{MSP+\left({n}_c-1\right) MSG} $$

where MSP is the mean square error within the populations, MSG represents the mean square error between the two populations, and *n*_*c*_ represents the average sample size of the entire population after correction [33, 34]. The EigenGWAS algorithm combines the statistical framework of genome-wide association studies with eigenvector decomposition to identify selection signatures on the underlying genome [16]. The EigenGWAS method uses the single marker regression model for association tests. However, its phenotype is different from the phenotype of typical GWASs and it is an individual-level eigenvector derived from genotype data. The model can be described as the following equation:

$$ {y}_{ki}=\mu +{b}_i{x}_{ij}+{e}_i $$

where *y*_*ki*_ is the *k* th eigenvector value of individual *j*; *x*_*ij*_ is the value of the *j* th SNP for individual *j*; *b*_*i*_ is the regression coefficient for the *i* th SNP. EigenGWAS can be used as a method to find the selection signatures among the population or across a gradient of ancestry. In this study, XPEHH, F_ST_, and EigenGWAS were used to detect selective footprints on the chicken Z chromosome. We used SHAPEIT (v2.12) software to generate haplotype data based on the SNPs data. We used the LD package of R (v3.6.1) to compute the XPEHH values. The threshold of the XPEHH at a significance level of 0.05 was ±2. Using VCFtools [35], the average F_ST_ value of all SNPs in each sliding window (window size: 100 Kb, step size: 10 Kb) was calculated. We determined the threshold for the outlier F_ST_ sliding windows average based on the following formula:

$$ \mathrm{Threshold}={Q}_3+1.5\times \left({Q}_3-{Q}_1\right) $$

Among them, *Q*_1_ is the lower quartile (first quartile); Q_3_ is the upper quartile (third quartile). In this study, the F_ST_ value of the sliding window greater than threshold (0.51) is defined as a selection signature. For EigenGWAS, EMMAX software [36] was used for single-marker regression. Threshold *P*-values of 0.05/1937 = 2.58 × 10^−5^ were used to confirm statistically significant differences. In this study, the candidate regions were determined within 1 Mb of each other regions identified by different methods.

To reveal the biological functions of the selection signatures, additional analyses were performed. We identified candidate genes within the selection regions using chicken gene annotation data from the Ensembl database. We then used the online software DAVID (v6.7) [11] to perform GO analysis based on the candidate genes obtained. Thirdly, selection regions were mapped onto QTL obtained from the chicken QTL database (https://www.animalgenome.org/cgi-bin/QTLdb/GG/index).

### Gene expression profiles

Gene expressions in the abdominal adipose tissue of seven-week-old NEAUHLF broilers were evaluated in chicken genome arrays. According to Wang et al. [37], the raw data set has been standardized using Affymetrix Microarray Suite 4.0 software and uploaded to the GEO database (GEO accession number: GSE8010). We downloaded the GSE8010 data set for subsequent gene differential expression analysis [37]. The ten birds were selected based on the percentage of abdominal fat (AFP) at 7-weeks for the G 8 of NEAUHLF broilers: the 5 chickens with the highest AFP in fat line and the 5 chickens with the lowest AFP in lean line. A one-way ANOVA was used to statistically compare the DEGs between fat and lean line chickens.

## Supplementary Information

**Additional file 1: Supplementary Table 1.**

## Data Availability

The SNP genotype data and the 7-week-old abdominal fat expression data of broilers used in this study were obtained from the Gene Expression Omnibus (GEO) database at the National Center for Biotechnology Information (NCBI) under accession numbers GSE58551 and GSE8010.

## Abbreviations

XPEHH: Cross Population Extend Haplotype Homozygosity Test
F_ST_: F-statistics
DEG: Differentially expressed genes
SNP: Single nucleotide polymorphism
NEAUHLF: Northeast Agricultural University broiler lines divergently selected for abdominal fat content
PCA: Principal component analysis
GO: Gene Ontology
QTL: Quantitative trait loci
TFM: Total fat mass
BMI: Body mass index
MAF: Minor allele frequencies
EHH: Extended haplotype homozygosity
AFP: Percentage of abdominal fat
